# Phenolics Profiling of *Carpobrotus edulis* (L.) N.E.Br. and Insights into Molecular Dynamics of Their Significance in Type 2 Diabetes Therapy and Its Retinopathy Complication

**DOI:** 10.3390/molecules26164867

**Published:** 2021-08-11

**Authors:** Saheed Sabiu, Fatai O. Balogun, Stephen O. Amoo

**Affiliations:** 1 Department of Biotechnology and Food Science, Durban University of Technology, P.O. Box 1334, Durban 4000, South Africa; balogunfo@yahoo.co.uk; 2 Agricultural Research Council—Vegetables, Industrial and Medicinal Plants, Pretoria, Private Bag X293, Pretoria 0001, South Africa; AmooS@arc.agric.za; 3 Indigenous Knowledge Systems Centre, Faculty of Natural and Agricultural Sciences, North-West University, Private Bag X2046, Mmabatho 2735, South Africa; 4 Department of Botany and Plant Biotechnology, Faculty of Science, University of Johannesburg, P.O. Box 524, Auckland Park 2006, South Africa

**Keywords:** aldose reductase, alpha-amylase, alpha-glucosidase, enzyme inhibitors, molecular dynamics simulation, phenolics

## Abstract

Adverse effects associated with synthetic drugs in diabetes therapy has prompted the search for novel natural lead compounds with little or no side effects. Effects of phenolic compounds from *Carpobrotus edulis* on carbohydrate-metabolizing enzymes through in vitro and in silico methods were assessed. Based on the half-maximal inhibitory concentrations (IC_50_), the phenolic extract of the plant had significant (*p* < 0.05) in vitro inhibitory effect on the specific activity of alpha-amylase (0.51 mg/mL), alpha-glucosidase (0.062 mg/mL) and aldose reductase (0.75 mg/mL), compared with the reference standards (0.55, 0.72 and 7.05 mg/mL, respectively). Molecular interactions established between the 11 phenolic compounds identifiable from the HPLC chromatogram of the extract and active site residues of the enzymes revealed higher binding affinity and more structural compactness with procyanidin (−69.834 ± 6.574 kcal/mol) and 1,3-dicaffeoxyl quinic acid (−42.630 ± 4.076 kcal/mol) as potential inhibitors of alpha-amylase and alpha-glucosidase, respectively, while isorhamnetin-3-*O*-rutinoside (−45.398 ± 4.568 kcal/mol) and luteolin-7-*O*-beta-d-glucoside (−45.102 ± 4.024 kcal/mol) for aldose reductase relative to respective reference standards. Put together, the findings are suggestive of the compounds as potential constituents of *C. edulis* phenolic extract responsible for the significant hypoglycemic effect in vitro; hence, they could be exploited in the development of novel therapeutic agents for type-2 diabetes and its retinopathy complication.

## 1. Introduction

Diabetes, one of the leading causes of death globally, is a chronic metabolic derangement leading to high levels of glucose in systemic circulation (hyperglycaemia) due to the inability of the body to manage available glucose levels, arising from ineffective or insensitive insulin secretion by islets of Langerhans beta cells of the pancreas [1]. The management of the disease is time-consuming, during which diverse secondary complications (nephropathy, neuropathy, cardiopathy, retinopathy, etc.) culminating in death may set in, if no viable treatment/management therapy is embraced. The prevalence of diabetes continues to rise with population growth, and alongside other non-communicable diseases accounted for 74% of the world’s total deaths in 2019 [2]. Uncontrolled hyperglycaemia stimulates excessive free radical generation, culminating in oxidative stress associated with most diabetic complications such as neuropathy, nephropathy and retinopathy [3].

Glycaemic control remains one of the therapeutic approaches to hyperglycaemia and the risk of developing complications, and this has been achieved through lifestyle modification and conventional oral hypoglycaemic drugs [4]. Although drugs such as acarbose and other α-glucosidase inhibitors have been identified as therapeutic agents against the key enzymes implicated in carbohydrate metabolism in the gastrointestinal tract, the associated adverse effects as evident in increased postprandial blood glucose level in diabetics have undermined their application [5]. The occurrence of adverse effects is also consistent with synthetic inhibitors of aldose reductase, a major enzyme of the polyol pathway and a drug target in the clinical treatment of retinopathy complication of type 2 diabetes mellitus [6]. To this end, the pharmacological use of plant-derived phenolics with proven antioxidant potentials has been recognized as an important strategy in both glycaemic control and the management/treatment of diabetic complications such as retinopathy [7].

Phenolic compounds have beneficial health-promoting properties and are regarded as the class of compounds mostly found in edible plants, fruits, and beverages among the phytochemicals [8]. They are effective in the treatments against several debilitating diseases such as cancer, diabetes, inflammation, pathogenic infections and aging, amongst others [9,10]. While some phenolics have demonstrated potential in the treatment and management of diabetes and its complications [11], an insight into deciphering the nature of interaction(s) with the implicated key enzymes is imperative to provide evidence-based data supporting their antidiabetic potential.

*Carpobrotus edulis* (L.) N.E.Br. is a robust, flat-growing plant belonging to the family Aizoceae. The genus Carpobrotus comprises 14 species [12] with seven of them (*C. acinaciformis, C. deliciosus, C. mellei, C. dimidiatus, C. quadrifidus, C. edulis* and *C. muirii*) endemic to southern Africa [13]. *Carpobrotus edulis* is locally referred to as Cape Fig (English), umgongozi (Zulu), perdevy, ghaukum, suurvy, ghoenavy, Hottentotsvy, Kaapsevy, rankvy, vyerank (Afrikaans) and is naturalized in Australia (west coast), England and some parts of the Mediterranean, though widely distributed within the capes of South Africa [13]. Juice from its leaves is ethnomedicinally used to treat dysentery, stomach cramps, sore throat, mouth infections, burns, bruises, scrapes, wounds, skin ailments (ringworm, dermatitis, eczema, sunburn, nappy rash herpes, cold sores, allergies) as well as tuberculosis (when mixed with either honey or olive oil) [10,13]. Besides its application in maintaining healthy pregnancy, formulations from the plant have also been reported to be therapeutic against diphtheria and diabetes [13].

Despite its acclaimed ethnobotanical use against diabetes [13] and a report on the antidiabetic potential of its extract linked to its phenolic constituents [14], the exact mechanism and/or nature of its interaction(s) with the key enzymes linked with carbohydrate metabolism/type 2 diabetes, which accounts for over 90% of diabetes cases, remains elusive. Hence, for the first time, this study provided computational insights into the mechanism of modulatory effect of its phenolics on the activity of α-amylase and α-glucosidase while reporting its role in diabetic retinopathy complication.

## 2. Results and Discussion

### 2.1. Phenolic Content

**Figure 1:**
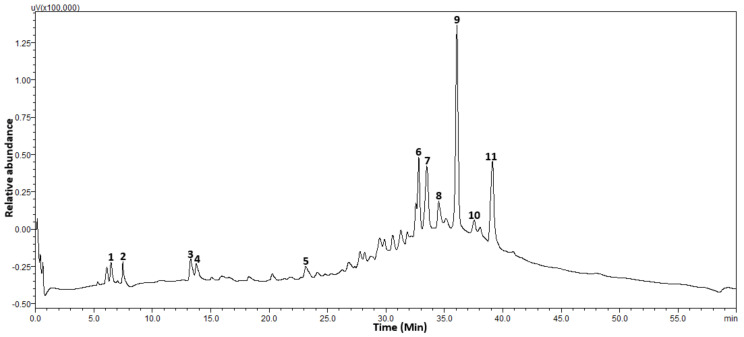
HPLC chromatogram of *Carpobrotus edulis*. **1**: Sinapic acid, **2**: Cacticin, **3**: Hyperoside, **4**: 1,3Dicaffeoylquinic acid, **5**: Procyanidin, **6**: Rutin, **7**: Epicatechin, **8**: Isorhamnetin-3-*O*-rutinoside, **9**: Chlorogenic acid, **10**: Myricetin, **11**: Luteolin 7-*O*-beta-d-glucoside.

The total phenolic content of the extract was 96.05 mg/g GAE. Based on the standards used for the HPLC analysis, 11 major phenolic compounds including sinapic acid, cacticin, hyperoside, 1,3-dicaffeoxyl quinnic acid, procyanidin, luteolin-7-*O*-beta-d-glucoside, rutin, epicatechin, isorhamnetin-3-*O*-rutinoside, chlorogenic acid and myricetin were identified from the chromatogram of the extract (Figure 1, Table 1). It is noteworthy that the prominent peaks as observed from the chromatogram with their highest relative abundances were for chlorogenic acid followed by rutin, luteolin-7-*O*-beta-d-glucoside and epicatechin, suggesting they are the major identifiable phenolic constituents of *C. edulis* (Figure 1).

### 2.2. Antidiabetic Activity

The antidiabetic effect of phenolic extract of *C. edulis* based on its half-maximal inhibitory concentrations (IC_50_) of 0.06 and 0.75 mg/mL revealed significantly (*p* < 0.05) higher inhibitory activity against alpha-glucosidase and aldose reductase, respectively, than the standards [acarbose (0.72 mg/mL) and ranirestat (7.05 mg/mL)], except for alpha-amylase, where the extract (0.51 mg/mL) favourably competed with acarbose (0.55 mg/mL) (Table 2). Alpha-amylase and alpha-glucosidase found in the pancreas and intestine, respectively, are prominent enzymes involved in the uncontrolled or unending hydrolysis of carbohydrate to glucose. In a diabetic state, the control (with inhibitors) of these enzymes is necessary for the management of hyperglycaemia. Due to the available synthetic inhibitors such as acarbose and miglitol triggering gastro-intestinal dysfunctional events including bloating, flatulence and diarrhoea, the use of plant-based alternative therapeutic agents has been advocated [15], provided they are able to strongly (low IC_50_ score) and mildly (marginally high IC_50_ value) inhibit the specific activity of alpha-glucosidase and alpha-amylase [16,17], as observed in this study with *C. edulis* phenolic extract. Although in a related study [14], among the studied extracts (50% methanol, 70% acetone and aqueous) of the plant, only aqueous extract was tested against alpha-glucosidase, the result obtained in the current study fared comparably in terms of activity with the previously determined aqueous extract, which not only corroborates the antihyperglycemic effect of the plant but the use of polar solvent as medium of extraction or herbal preparation in indigenous medicine. Overall, the exhibited antihyperglycemic effect by the extract could be attributed to its phenolic compounds, as studies have reported phenolic compounds such as cacticin, epicatechin, luteolin glucosides, cyanidin (identified in this study) in plants such as *Bergenia ciliata*, *Diospyros kaki* and *Eleusine coracana* as inhibitors of alpha-amylase and alpha-glucosidase [7,18]. Aldose reductase, an enzyme implicated in the complications of diabetes, particularly retinopathy, facilitates sorbitol production from glucose through polyol pathway during the glucose catabolism [19]. Drugs such as ranirestat, sorbinil and alrestatin act by improving the reduced glucose concentration, thus facilitating eventual absorption in tissues (such as neural, lens and glomeruli) [20]. However, like many other synthetic drugs, they exhibit side effects including nausea, fever and diarrhoea, hence, the need for alternative therapeutic molecules (inhibitors) from natural products in drug design. Based on its IC_50_ value with respect to modulatory effect of inhibitors on aldose reductase, the elicited activity by *C. edulis* phenolic extract in this study could be indicative of its prospect in the management of long-term diabetic retinopathy complication [21,22,23,24,25]. Patel et al. [26] also advocated phenolic compounds such as luteolin-7-*O*-β-d-glucopyranoside and 4,5-di-*O*-caffeoylquinic acid which are similar compounds identified in this study, as inhibitors of aldose reductase with prospect for diabetes retinopathy.

### 2.3. Molecular Docking and Dynamics

To gain insight into the probable interactions between the identified phenolic compounds (as revealed by the HPLC analysis) and the study enzymes in this study, computational evaluation was performed through molecular docking and MDS. Molecular docking, a measure of fitness and pose of a compound at the active site of an enzyme, normally gives high negative scores as a reflection of better pose of the compound [27]. In this study, phenolic compounds such as 1,3-dicaffeoxyl quanic acid, chlorogenic acid, epicatechin, luteolin-7-*O*-beta-d-glucoside, isorhamnetin-3-*O*-rutinoside, myricetin and rutin, had significant and better poses based on their scores than the reference standard, ranirestat, when docked with aldose reductase (Table 3). Additionally, better poses were observed for epicatechin, luteolin-7-*O*-beta-d-glucoside, isorhamnetin-3-*O*-rutinoside, rutin, hyperoside and procyanidin with alpha-amylase compared to the resulting complexes with acarbose (Table 3). While most of the identified compounds including 1,3-dicaffeoxyl quanic acid, chlorogenic acid, epicatechin, isorhamnetin-3-*O*-rutinoside, luteolin-7-*O*-beta-d-glucoside, myrcetin, rutin, cacticin, hyperoside and procyanidin showed good docking with alpha-glucosidase as depicted by the higher negative values than acarbose, other compounds (epicatechin, isorhamnetin-3-*O*-rutinoside, chlorogenic acid and rutin) had commendable binding at the active sites of the three enzymes (Table 3), which is indicative of their prospective interaction with the enzymes [28]. However, since docking is only a preliminary reflection of the ligand’s fitness within the binding pocket of a receptor, the binding orientations of the studied phenolics were subjected to further binding energy calculations and MDS. Typically looking at the thermodynamic calculations against alpha-amylase, procyanidin among other compounds had the highest (−69.834 kcal/mol) binding energy, which was better than the value for acarbose (−54.679 kcal/mol) and rutin (−46.826 kcal/mol) (Table 4). Similarly, against alpha-glucosidase, 1,3-dicaffeoxyl quinic acid and hyperoside had higher binding energies than acarbose, while isorhamnetin-3-*O*-rutinoside by luteolin-7-*O*-beta-d-glucoside and rutin had higher binding energies than ranirestat against aldose reductase (Table 4). Higher negative values are indicative of stronger affinity of these compounds with the respective enzymes and hence possible better stability of the resulting complex [29]. While potential stronger affinities of phenolic compounds (over synthetic inhibitors) in complex with antidiabetic enzymes have been reported [30,31], Rasouli et al. [18] observed higher binding free energy of some of the phenolic compounds (rutin, cacticin and epicatechin) reported in this study against alpha-amylase as well as alpha-glucosidase.

**Figure 2:**
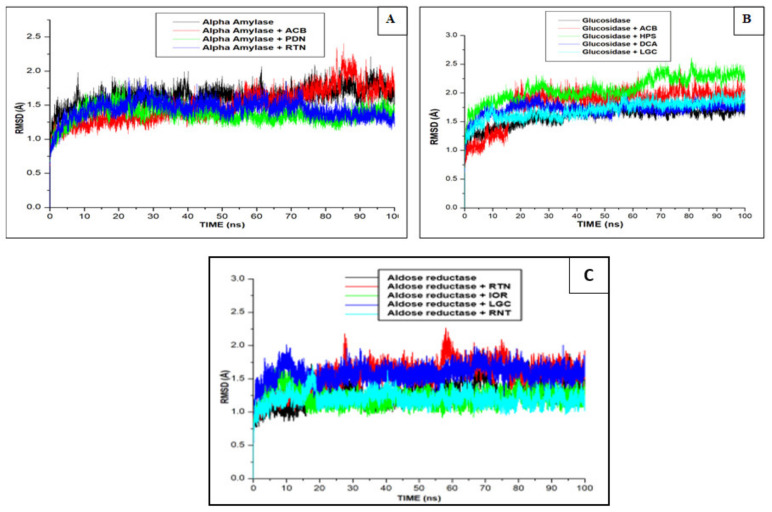
Comparative plots of α-carbon of (**A**) alpha-amylase, (**B**) alpha-glucosidase and (**C**) aldose reductase and phenolic compounds and standard molecules (acarbose, ranirestat) presented as RMSD determined over 100 ns molecular dynamics simulations. ACB: Acarbose; RNT: Ranirestat; PDN: Procyanidin; RTN: Rutin; HPS: Hyperoside; DCA: 1,3-Dicaffeoxyl quinic acid; IOR: Isohamnetin-3-*O*-rutinoside; LGC: Luteolin7-*O*-beta-d-glucoside.

**Figure 3:**
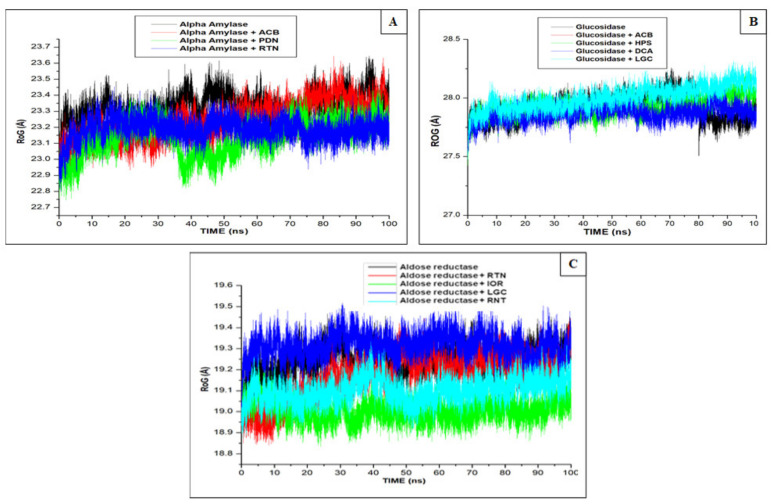
Comparative plots of α-carbon of (**A**) alpha-amylase, (**B**) alpha-glucosidase, and (**C**) aldose reductase, phenolic compounds and standard molecules (acarbose, ranirestat) presented as RoG determined over 100 ns molecular dynamics simulations. ACB: Acarbose; RNT: Ranirestat; PDN: Procyanidin; RTN: Rutin; HPS: Hyperoside; DCA: 1,3-Dicaffeoxyl quinic acid; IOR: Isohamnetin-3-*O*-rutinoside; LGC: Luteolin7-*O*-beta-d-glucoside.

**Figure 4:**
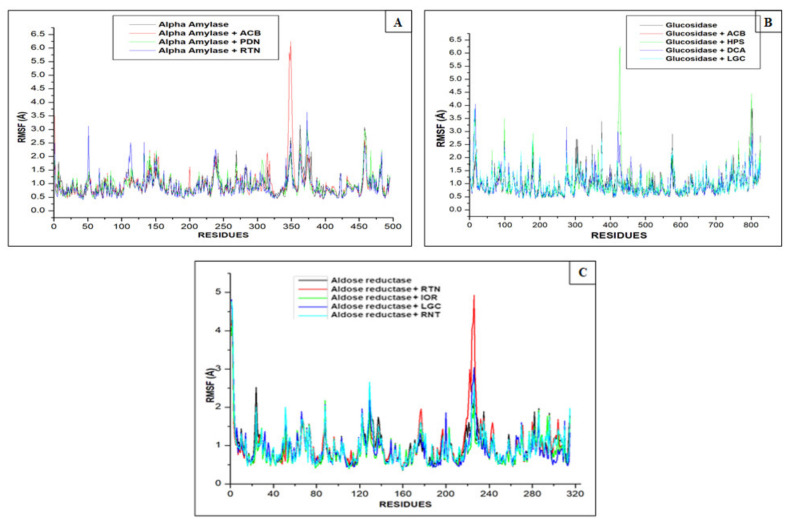
Comparative plots of α-carbon of (**A**) alpha-amylase, (**B**) alpha-glucosidase and (**C**) aldose reductase and phenolic compounds and standard molecules (acarbose, ranirestat) presented as RMSF and determined over 100 ns molecular dynamics simulations. ACB: Acarbose; RNT: Ranirestat; PDN: Procyanidin; RTN: Rutin; HPS: Hyperoside; DCA: 1,3-Dicaffeoxyl quinic acid; IOR: Isohamnetin-3-*O*-rutinoside; LGC: Luteolin7-*O*-beta-d-glucoside.

The enzyme-ligand complex is prone to conformational changes inducible by the binding ligand, and this could result in possible alteration of the biological activity of the enzyme [31]. Based on the affinity of the enzymes for the phenolics through 100 ns MDS in this study, a further probe into understanding the degree of stability and compactness of the resulting complexes was undertaken. To examine the stability while ensuring the equilibration of the enzyme-ligand, parameters including RMSD, RMSF and RoG evaluated over an extended period of 100 ns for unbound (apo-enzyme, i.e., alpha-amylase, alpha-glucosidase and aldose reductase) and bound complexes (enzyme + inhibitor, i.e., standards and phenolic compounds) are presented in Figure 2, Figure 3, Figure 4.

The nature/extent of stability and convergence of a ligand-receptor system is normally measured by RMSD [28]. In this study, the average RMSD values for procyanidin, rutin and acarbose (standard drug) were 1.62 Å, 1.68 Å and 1.82 Å, respectively, while it was 2.04 Å for α-amylase (apo-enzyme) (Figure 2A). With these findings, RMSD of the compounds and standard were lower than the apo-enzyme, meaning that both the compounds and standard possessed the ability to promote increased structural stability of the alpha-amylase. Additionally, the lower RMSD of the phenolic compounds compared to the standard is suggestive of their better advantage as prospective leads and possible novel inhibitors of the enzyme. Figure 2B shows higher average RMSD bound systems for the phenolic compounds, hyperoside (2.22 Å), 1,3-dicaffeoxyl quinic acid (1.73 Å) and luteolin-7-*O*-beta-d-glucoside (1.70 Å) relative to the apo-enzyme, alpha-glucosidase (1.48 Å). However, it must be noted that these compounds, particularly 1,3-dicaffeoxyl quinic acid and luteolin-7-*O*-beta-d-glucoside, had RSMD values that are not only within acceptable limits, but lower than that of the standard, acarbose (1.79 Å), pointing to their relative superiority over the reference standard. Interestingly, these results are in tandem with the thermodynamic profiles where 1,3-dicaffeoxyl quinic acid had the best binding free energy (Table 4), synonymous with better compactness and structural stability of the complex. A similar trend was also observed where the RMSD value of the apo-enzyme was lower than the bound systems regarding aldose reductase. Typically, aldose reductase average RMSD (1.32 Å) was lower than ranirestat system (1.38 Å) which was not significant but only marginally lower than the value (1.43 Å) observed with luteolin-7-*O*-beta-d-glucoside (Figure 2C). This observation may not infer the inability of luteolin-7-*O*-beta-d-glucoside to promote the structural stability of the complex going by comparable RMSD value with ranirestat. Aside from the fact that the value is within acceptable limit, the effect elicited by luteolin-7-*O*-beta-d-glucoside with respect to its RMSD value corroborates its enhanced binding free energy in comparison with other compounds and the reference standard (Table 4).

Radius of gyration (RoG) determines the total compactness of the enzyme-inhibitor binding [28,32]. It is a measure of densification of the protease structure [33], and the smaller the RoG value, the better the protease stability. In line with RMSD result, the lead compounds and standard drug revealed mean RoG values of 23.24 Å (procyanidin), 23.25 Å (rutin) and 23.37 Å (acarbose) lower than the apo-enzyme (23.54 Å), indicating that the binding of the compounds potentially stabilized alpha-amylase better than the control molecule (Figure 3A). However, the RoG results of compounds and standard drugs for alpha-glucosidase and aldose reductase do not follow the trend of RMSD, as there were reductions in RoG values of phenolic compounds such as 1,3-dicaffeoxyl quinic acid (27.64 Å), hyperoside (27.78 Å) and the standard, acarbose (27.78 Å), when compared with alpha-glucosidase (27.81 Å), except luteolin-7-*O*-beta-d-glucoside (28.23 Å) (Figure 3B). A similar pattern to unbound apo-enzyme (alpha-glucosidase) was observed with aldose reductase (19.27 Å) where isorhamnetin-3-*O*-rutinoside (18.97 Å), rutin (19.26 Å) and acarbose (19.22 Å), except luteolin-7-*O*-beta-d-glucoside (19.40 Å), had higher RoG values (Figure 3C).

The binding property of the inhibitor or ligand and the active site residues of each protein was further evaluated by RMSF. Increased or decreased fluctuations are sin qua non to high or low flexibility movement or interaction between ligands and the receptor amino acids residues [28]. In the finding for alpha-amylase system, rutin (2.79 Å) followed by acarbose (2.54 Å) exhibited the highest average RMSF values, while the lowest value was found with procyanidin (2.05 Å) among the studied interactions. While it was observed that compounds and the standard drug increased the enzyme (1.90 Å) fluctuation or amino acid residue flexibility, a kind of similar pattern of fluctuations was seen among the compounds, the standard drug and enzyme at 200, 325 and 350 residues (Figure 4A). Except for luteolin-7-*O*-beta-d-glucoside (1.88 Å), compounds including hyperoside (4.31 Å) and 1,3-dicaffeoxyl quinic acid (3.24 Å) were found to have higher average RMSF above the enzyme (3.06 Å). The observed fluctuations were seen around 350, 425 and 800 residues (Figure 4B). The highest RMSF in the aldose reductase system was 2.88 Å (standard drug), while the lowest for the studied interactions was 1.28 Å (isorhamnetin-3-*O*-rutinoside). The compounds, especially isorhamnetin-3-*O*-rutinoside and luteolin-7-*O*-beta-d-glucoside (1.45 Å), were able to reduce the fluctuation of the enzyme having an RMSF of 1.85 Å. The fluctuations occurred at 180 and 220 of the amino acids’ residues (Figure 4C).

**Figure 5:**
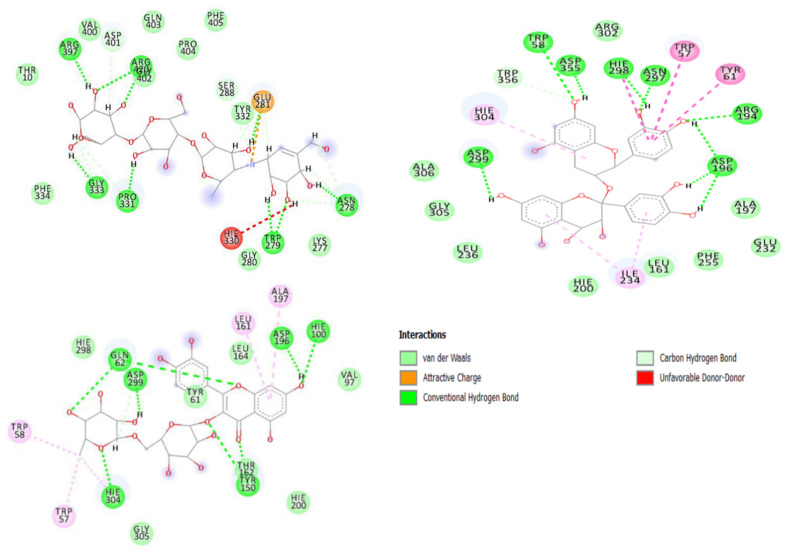
Interaction types and plots between phenolic compounds, standard drug and alpha-amylase. (**A**): Acarbose (ACB); (**B**): Procyanidin (PDN); (**C**): Rutin (RTN).

**Figure 6:**
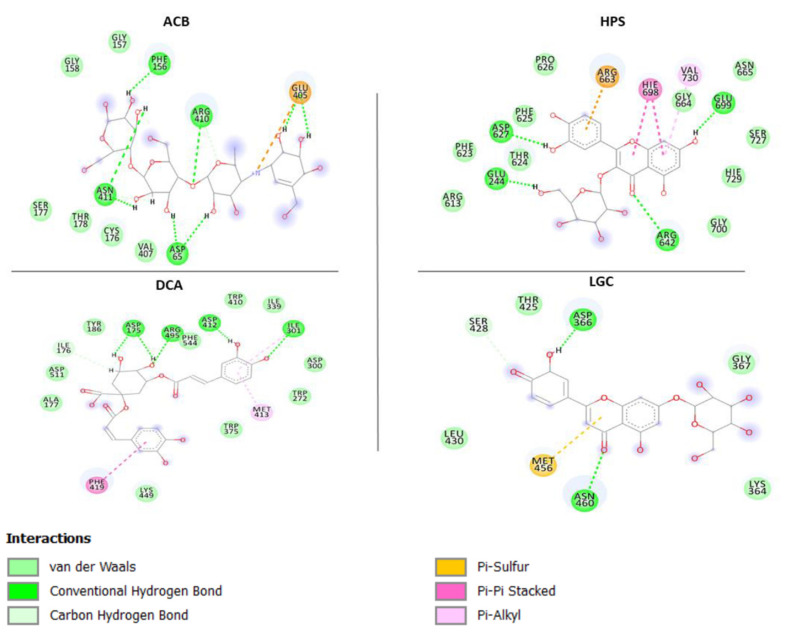
Interaction types and plots between phenolic compounds, standard drug and alpha-glucosidase. ACB: Acarbose; HPS: Hyperoside: PDN; DCA: 1,3-Dicaffeoxyl quinic acid.

**Figure 7:**
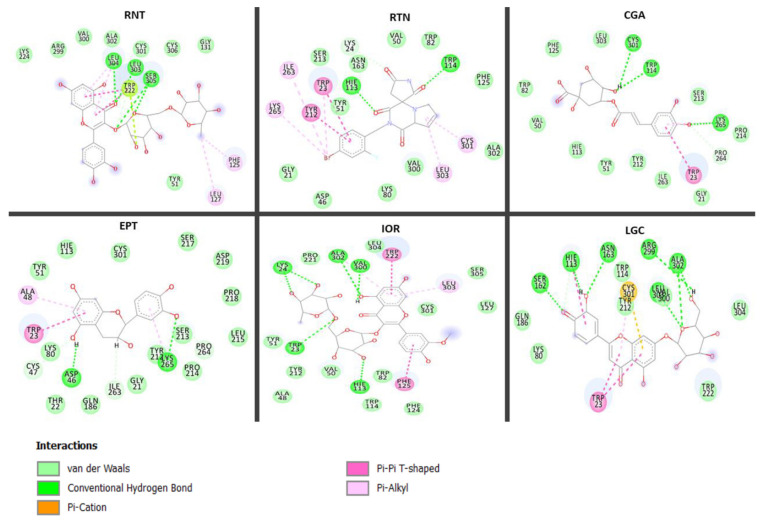
Interaction types and plots between phenolic compounds, standard drug and aldose reductase. RNT: Ranirestat; RTN: Rutin; IOR: Isohamnetin-3-*O*-rutinoside; LGC: Luteolin7-*O*-beta-d-glucoside; CGA: Chlorogenic acid; EPT: Epicactin.

The interaction between the binding of molecules (ranirestat, acarbose) or compounds with the active site residues of the enzymes (alpha-amylase, alpha-glucosidase and aldose reductase) is represented by ligand-enzyme interaction plots (Figure 5, Figure 6, Figure 7). The interactions between acarbose (standard), procyanidin and rutin on the active sites of alpha-amylase from the plots (Figure 5A–C) were Van der Waals forces, hydrogen (to hydrogen) bonds, donor-donor interaction, C–H bond, π-π stacked interaction and π-alkyl bonds, though the number of these interactions differs between molecules and observed to be a consequence of their binding free energies. While acarbose Van der Waals forces (with Gln403, Phe405, Val400, Pro404, Thr332, Thr10, Phe334), H–H bonds (Arg397, Arg420, Gly333, Pro331, Trp279, Asn278) and C-H (Asp401, Lys277, Gly280, Ser288) formed, it had one interaction fewer compared with procyanidin (21 bonds) [Van Der Waal (Arg302, Ala306, Gly305, Leu236, Hie200, Leu161, Phe255, Glu232, Ala197), H–H bonds (Asp299, Asp196, Arg194, Trp58, Asp355, Hie298, Asn297), π-π (Trp57, Tyr61) and π-alkyl (Hie304, Ile234)], and this could explain why it had lower binding free energy in comparison with procyanidin (Table 4), which possessed more numbers of hydrogen bonds and presence of π-π stacked interaction and π-alkyl bonds. The binding free energy capacity of rutin (lower than acarbose and procyanidin) is corroborated by its number of molecular interactions [(17) including Van Der Waals (Hie298, Hie200, Tyr61, Gly305, Leu164, Val97), H–H bonds (Gln62, Asp299, Asp196, Hie100, Hie304, Tyr150) and π-alkyl bonds (Leu161, Ala197, Trp58, Trp57)]. In terms of amino acid residues involved in the stability, it was observed that Trp57, Trp58, Try61, Leu162, Asp196, His201, Asp299 and Ala197 are the most important amino acid residues involved with compounds (procyanidin and rutin) at the active sites of alpha-amylase. Though these residues are absent in acarbose, our report agrees with the submission of Hashim et al. [34], where Trp57, Trp58 and His201 have also been identified as important (catalytic) residues involved in alpha-amylase (1DHK) stability. 1,3-Dicaffeoxyl quinic acid [(Ala177, Asp511, Tyr186, Phe544, Tyr410, Ile339, Asp300, Trp272, Trp375, Lys449), (Asp175, Arg475, Asp412, Ile301) (Phe419), (Met413)] and hyperoside [(Arg613, Phe623, Phe625, Thr624, Pro626, Gly700, Gly664, Asn665, Ser727, Hie729), (Asp627, Glu244, Glu699, Arg642), (His698), (Val730) had the same number of interactions (17) with the active sites of alpha-glucosidase and are characterized by (contain the same number of) Van der Waal forces (10), H-bonds (4), π-π stacked interaction (1) and π-alkyl bonds (1); however the highest binding free energy found with 1,3-dicaffeoxyl quinnic acid may be attributed to unidentified carbon–H bonds (Ile176) and formed π-cation (Arg663) in hyperoside. In fact, the presence of π-cation in hyperoside may also be suggested to be the reason for lesser binding energy, as similarly witnessed in acarbose (Glu405) with far less binding energy and lacking π-π stacked, π-alkyl bonds and a lower number of Van der Waal forces (Gly157, Gly158, Ser177, Thr178, Cys176, Val407) (Figure 6). Similarly, the interactions [H-bonding (Leu303, Leu304, Leu305), vVn Der Waal forces (Lys224, Arg299, Val300, Ala302, Cys301, Cys306, Gly131, Tyr51), π-sulfur (Trp222), π-Alkyl (Phe125, Leu127) of ranirestat and standard molecule (14) with active sites of aldose reductase is lesser than those of isorhamnetin-3-*O*-rutinoside, rutin and luteolin-7-*O*-beta-d-glucoside exhibited in terms of number of interactions (20, 20 and 15 respectively) relative to the former (Figure 7), and these interactions corroborated the findings from the binding free energies (Table 4). It is interesting to note that although isorhamnetin-3-*O*-rutinoside and rutin revealed same number of interactions (20), the presence of higher numbers of Van der Waal forces [(12) (Pro221, Leu304, Cys301, Ser305, Leu127, Tyr51, Tyr212, Ala48, Val50, Trp82, Phe124, Trp114)], hydrogen bonds [(5) (Lys24, Ala302, Val300, Trp23, Hie113)] and absence of π-cation bond for isorhamnetin-3-*O*-rutinoside as against 11 (Ser213, Val50, Trp82, Asn163, Phe125, Tyr51, Ala302, Val300, Lys80, Gly21, Asp46), 2 (Hie113, Trp114) and 1 (Lys24), respectively, for rutin could be responsible for the higher binding free energy witnessed in the former over the latter. Amino acids residues such as Leu303, Leu304, Cys301, Cys306, Val300, Phe125, Trp22, Ser305, Tyr51, Ala302 and Arg299 were found surrounding acarbose. While some of these amino acids residues, including Leu303, Cys301, Val300, Ala302 might be believed to be involved in the stability of the ligands-enzyme complexes because they are also shared by the promising compounds, others like Leu304 (rutin), Cys306 (all compounds), Tyr51, (luteolin-7-*O*-beta-d-glucoside), Phe125 (luteolin-7-*O*-beta-d-glucoside), Ser305 (luteolin-7-*O*-beta-d-glucoside) and Trp222 (rutin) are observed to be absent on the interaction plots of respective complexes. Additionally, and more importantly, the presence of π-π stacked interactions between amino acid residues [Trp23 and Tyr212 (rutin), Trp23 (luteolin-7-*O*-beta-d-glucoside) as well as Trp222 and Phe125 (isorhamnetin-3-*O*-rutinoside)] and aromatic rings on the compounds absent in ranirestat may be suggestive of its low free energy binding. The presence of π-π stacked interactions in receptor-ligand binding has been reported to be germane in the development of drugs [35]. Hence, it could be inferred that the presence of π-π stacked interaction in these compounds could be advantageous in the development of potential inhibitors against carbohydrate enzymes that trigger diabetes development and its related complication (retinopathy).

## *3.* Materials and Methods

Acarbose, ranirestat, porcine pancreatic α-amylase, rat intestine acetone powder, aldose reductase, extra pure starch, dinitrosalicylic acid (DNS), *p*-nitrophenyl glucopyranoside (*p*NPG) and 2-chloro-4-nitrophenyl α-d-maltotrioside were obtained from Sigma-Aldrich, St. Louis, MO, USA. All other chemicals and reagents used are of analytical grade. *Carpobrotus edulis* leaves collected from the Agricultural Research Council—Vegetables, Industrial and Medicinal Plants campus in Pretoria, South Africa with voucher specimen Mulaudzi RB# 200 deposited in Bews Herbarium, University of KwaZulu-Natal, as described by Mulaudzi et al. [14] were lyophilised and ground into fine powders using a rotor mill (Fritsh Pulverisette 14, Labotec, Midrand, South Africa).

### 3.1. Phenolic Extract Preparation and Quantification

The method of Mulaudzi et al. [14] with slight modification was adopted for the extraction of the powdered materials from the leaves of the plant (10 g) in 50% methanol (250 mL) under sonication in a cold water-containing water bath for 120 min. The extract was filtered through a Whatman No. 1 filter paper, centrifuged (3000 rpm, 15 min), and the resulting supernatant concentrated in a water bath at 40 °C. The dried extract obtained was kept airtight and refrigerated (10 °C) in a glass vial prior to use for total phenolic quantification, HPLC analysis and in vitro antidiabetic assays.

For total phenolic quantification, 50 µL (1 mg/mL) of the phenolic extract was added to 6.950 mL distilled water in a test tube, and gently shaken prior to the addition of Folin-Ciocalteau phenol reagent (0.5 mL) and sodium carbonate (1.5 mL; 20%). Subsequently, distilled water (1 mL) was added to the mixture, shaken vigorously and allowed to stand for 45 min prior to absorbance measurement at 760 nm. The standard (gallic acid) was prepared (0.8 mg/mL in 40% methanol) and its varying concentrations were treated in a similar manner as the phenolic extract [36]. The phenolic content of the extract was estimated from gallic acid standard curve and expressed as milligram per gram gallic acid equivalent (mg/g GAE).

### 3.2. HPLC Analysis

The HPLC analysis was achieved based on the method of Peng et al. [37] with modifications. This was conducted using HPLC (Shimadzu Prominence-i LC-2030C 3D plus, Kyoto, Japan) coupled to a diode array UV detector (HPLC-DAD) and a high-resolution mass spectrometry (HPLC-HRMS) on an Ultimate 3000 RSL Cnano system (Thermo Scientific, Waltham, Massachusetts, United States of America). The mobile phase consisted of A (0.1% formic acid) and B (acetonitrile), and the flow rate was 0.25 mL/min with the temperature of the column (Sunfire C18, 5 µm, 4.6 mm × 150 mm, Waters Corporation, Milford, Massachusetts, United States of America) set at 35 °C and the sample volume maintained at 20 µL. The elution gradient varied from 1% A to 2% B linearly for 2 min and from 2–100% B in 50 min and thereafter, from 10% to 2% for 1 min and from 2% to 0% for 9 min. The chromatogram was based on photo diode array UV detector (DAD) with wavelengths spanning 190–800 nm based on the peak absorption of the analysed compounds. The identification of the compounds was achieved based on their individual retention times and MS fragment patterns compared with those of the standard phenolics (sinapic acid, cacticin, hyperoside, 1,3-dicaffeoxyl quinic acid, procyanidin, rutin, epicatechin, isorhamnetin-3-*O*-rutinoside, chlorogenic acid, myricetin and luteolin-7-*O*-beta-d-glucoside) used in tandem with published data.

### 3.3. In vitro Assays

#### 3.3.1. Alpha-Amylase Inhibitory Assay

Using a previously reported protocol [38], the activity of the extract against α-amylase was evaluated. Aliquots (0.1 mL) of either acarbose (reference standard) or the phenolic extract at varying concentrations (0.065–1.000 mg/mL) were added to α-amylase solution (0.1 mL of 0.5 mg/mL). Following a 10-min pre-incubation (25 °C) of the resulting solution, 1% starch solution in 0.02 M sodium phosphate buffer, was added and further incubated (25 °C, 10 min), before the reaction was halted by DNS (0.5 mL). The resulting mixtures were boiled (100 °C, 5 min) and subsequently cooled (25 °C) before final dilution (distilled water, 7.5 mL) and spectrophotometric absorbance reading (540 nm) (OPTIZEN POP, Apex Scientific, Yuseong-gu, Daejeon, Republic of Korea). The results presented as IC_50_ (half-maximal inhibitory concentration) value in each case was non-linearly extrapolated from maltose standard calibration curve.

#### 3.3.2. Alpha-Glucosidase Inhibitory Assay

For this assay, 50 µL of varying concentrations (0.065–1.000 mg/mL) of either acarbose or phenolic extract were added to 0.1 mL α- glucosidase (1 M) before incubation (25 °C, 10 min). Thereafter, 0.05 mL of 5 mM *p*-NPG solution was added followed by a further 5 min incubation (25 °C) period before 0.05 mL Na_2_CO_3_ (0.1 M). Following this treatment, a microplate reader (MULTISKAN GO 1519 Thermo Scientific, Vantaa, Finland) was used to take the absorbance readings (405 nm) and the IC_50_ values were similarly non-linearly determined from a *p*-nitrophenol standard curve [38].

#### 3.3.3. Aldose Reductase Determination

In this assay, glyceraldehyde and NADPH were used as substrate and cofactor, respectively [39]. The final concentration of the dissolving solvent was kept equivalent among reaction mixtures in the presence of varying concentrations of either the phenolic extract or ranirestat (reference standard). The rates of reaction were monitored spectrophotometrically (340 nm) at 25 °C and compared with the control not containing the phenolic extract, and the IC_50_ value was non-linearly determined from a calibration curve. Ranirestat was used as reference standard and the experiments were performed in triplicate.

### 3.4. In Silico Analysis

The collection of crystal (X-ray) structure of the enzymes [PDB: 3RX3 (aldose reductase), 3W37 (α-glucosidase), and 1DHK (α-amylase)] were from the RSCB Protein Data Bank (https://www.rcsb.org/ accessed on 12 December 2020). The UCSF Chimera software V1.14 was used in the preparation of the enzymes in readiness for docking [40], PubChem (https://pubchem.ncbi.nlm.nih.gov/ accessed on 15 December 2020) was used to retrieve the structures of the chromatogram-identified phenolic compounds (sinapic acid, cacticin, hyperoside, 1,3-dicaffeoxyl quinic acid, procyanidin, rutin, epicatechin, isorhamnetin-3-*O*-rutinoside, chlorogenic acid, myricetin and luteolin-7-*O*-beta-d-glucoside) and standards (acarbose and ranirestat) and optimization of their three-dimensional structures executed using Avogadro software as previously reported [41]. The optimized compounds (ligands) and the enzymes were subsequently subjected to molecular docking.

The docking of the prepared phenolic compounds and standards into binding pockets of the enzymes (α-amylase, α-glucosidase, and aldose reductase) was by Autodock Vina Plugin on Chimera V1.14. Judging by the docking scores, complexes identified to have the best pose for each compound were ranked, selected and further analyzed through 100 ns molecular dynamics simulation (MDS).

The MDS was achieved as recently reported [28], using the GPU (force fields) version obtainable in AMBER package, where the description of the system by FF18SB variant of the AMBER force field was carried out [42]. With the aid of Restrained Electrostatic Potential (RESP) and the General Amber Force Field (GAFF) methods of the ANTECHAMBER assisted with information on atomic partial charges for the compounds. Hydrogen atoms and Na+ and Cl- counter ions (to neutralize the system) were made possible with Leap module of AMBER 18. The residues were numbered 1–336, 913, and 496, respectively, for aldose reductase, α-glucosidase and α-amylase. The system in each case was then lowered implicitly within an orthorhombic box of TIP3P water molecules such that all atoms were within 8Å of any box edge. MDS total time carried-out were 100 ns. For each simulation, hydrogens atoms were constricted using the SHAKE algorithm. The step size of each simulation was 2 fs, and an SPFP precision model was used. The simulations align with the isobaric-isothermal ensemble (NPT), having randomized seeding, Berendsen barostat maintains 1 bar constant pressure, 2 ps pressure-coupling constant, 300 K temperature and Langevin thermostat with a collision frequency of 1.0 ps [43].

Using PTRAJ, the systems were subsequently saved, and each trajectory analyzed every 1 ps, and the RoG, RMSF, and RMSD were analyzed with CPPTRAJ module (AMBER 18 suit).

Molecular Mechanics/GB Surface Area method (MM/GBSA) was adopted to assess the free binding energy while comparison of the systems binding affinity followed afterwards [44]. Binding free energy was averaged over 100,000 snapshots extracted from the 100 ns trajectory. The ΔG for each system (enzyme, complex and phenolics) was estimated as earlier reported [45].

### 3.5. Statistical Analysis

For the in vitro experiments, data analyses were carried out by Graph pad Prism version 3.0 using *t*-test (and nonparametric tests), supplemented with Mann–Whitney test. Results are expressed as mean ± standard error of the mean (SEM). Except otherwise stated, the raw data plots for the in silico evaluations were generated using the Origin data analysis software V18 (OriginLab, Northampton, MA, USA) (Seifert, 2014).

## 4. Conclusions

While the in vitro studies result gave an insight into possible antidiabetic potential of *C. edulis*, the HPLC analysis suggested and identified 11 phenolic compounds, which were further analysed as probable hypoglycaemic candidates through in silico studies. Based on the findings from the binding free energy, structural stability and compactness in this study, procyanidin was a better inhibitor of alpha-amylase, 1,3-dicaffeoxyl quinic acid against alpha-glucosidase while luteolin-7-*O*-beta-d-glucoside showed good inhibitory potentials of aldose reductase among other phenolic compounds. Thus, these molecules could be exploited in developing novel therapeutic candidates against postprandial hyperglycaemia and diabetic retinopathy.

## Figures and Tables

**Table 1:** HPLC-MS profiling of phenolics from *Carpobrotus edulis.*

Peaks	Retention Time (min)	*m/z* [M − H]^−^	Compound
1	6.8	223.09	Sinapic acid
2	7.7	477.35	Cacticin
3	13.2	463.27	Hyperoside
4	13.8	515.44	1,3-Dicaffeoylquinic acid
5	23.2	593.50	Procyanidin
6	33.0	609.45	Rutin
7	33.7	289.08	Epicatechin
8	34.5	623.56	Isorhamnetin-3-*O*-rutinoside
9	36.1	353.75	Chlorogenic acid
10	37.4	317.19	Myricetin
11	39.1	447.13	Luteolin 7-*O*-beta-d-glucoside

*m*/*z*: mass-to-charge ratio for each monodeprotonated phenolic compound.

**Table 2:** Inhibitory effect of phenolic extract of *Carpobrotus edulis* on carbohydrate metabolizing enzymes and aldose reductase.

Extract/Compound	Concentration (mg/mL)		
	Alpha-Amylase	Alpha-Glucosidase	Aldose Reductase
*C. edulis*	0.51 ± 0.07 ^a^	0.06 ± 0.01 ^a^	0.75 ± 0.05 ^a^
Acarbose	0.55 ± 0.09 ^a^	0.72 ± 0.05 ^b^	NA
Ranirestat	NA	NA	7.05 ± 0.05 ^b^

Values are expressed as mean ± standard error of the mean (SEM) of triplicate determinations. ^a,b^ Values bearing different superscripts within the same column for each parameter are different significantly (*p* < 0.05). NA = Not applicable.

**Table 3:** Molecular docking scores (kcal/mol) of the phenolic compounds with carbohydrate hydrolyzing enzymes and aldose reductase.

Compounds	Aldose Reductase	α-Amylase	α-Glucosidase
Acarbose (ACB)	ND	−7.7	−7.5
Ranirestat (RNT)	−8.4	ND	ND
1,3-Dicaffeoylquinic acid (DCA)	−9.7	−7.7	−8.1
Cacticin (CCT)	−7.9	−8.1	−7.6
Chlorogenic acid (CGA)	−8.9	−7.4	−7.6
Epicatechin (EPT)	−9.6	−8.4	−7.7
Hyperoside (HPS)	−7.5	−7.9	−8.4
Isorhamnetin-3-*O*-rutinoside(IOR)	−8.9	−8.8	−8.4
Luteolin 7-*O*-beta-d-glucoside (LGC)	−9.4	−9.2	−8.6
Myricetin (MYC)	−8.8	−7.7	8.1
Procyanidin (PDN)	−8.3	−8.6	−7.9
Rutin (RTN)	−8.9	−9.0	−7.8
Sinapic acid (SPA)	−6.6	−5.7	−6.4

ND: Not determined.

**Table 4:** Thermodynamic binding free energy profiles of the phenolic compounds and standard drugs with the study enzymes.

Complex	Energy Components (kcal/mol)				
	Δ E_vdW_	ΔE_elec_	ΔG_gas_	ΔG_solv_	ΔG_bind_
α-Amylase					
ACB	−52.578 ± 4.803	−93.386 ± 12.396	−145.965 ± 11.568	91.286± 9.321	−54.679 ± 4.890
CCT	−42.042 ± 4.060	−48.401 ± 2.379	−90.443 ± 12.273	48.248 ± 5.903	−42.195 ± 8.858
PDN	−45.013 ± 5.091	−111.131 ± 15.036	−156.145 ± 13.931	86.310 ± 9.183	−69.834 ± 6.574
RTN	−43.268 ± 4.016	−65.640 ± 5.205	−108.908± 12.001	62.081 ± 9.760	−46.826 ± 3.262
α-Glucosidase					
ACB	−24.164 ± 5.955	−396.679 ± 30.892	−420.843 ± 31.177	385.092 ± 23.859	−35.751 ± 9.641
CCT	−19.542 ± 4.245	−173.993 ± 21.584	−198.343 ± 23.812	162.521 ± 19.321	−30.857 ± 6.019
HPS	−32.536± 4.673	−65.783± 9.645	−98.319± 7.684	60.127± 12.513	−38.192± 6.407
DCA	−34.367 ± 4.263	−58.595 ± 11.108	−92.962 ± 9.421	50.331 ± 7.343	−42.630 ± 4.076
LGC	−21.894 ± 3.942	−183.993 ± 28.654	−205.887 ± 28.876	172.531 ± 23.163	−33.355 ± 7.119
RTN	−24.254 ± 1.113	−55.254 ± 5.548	−87.478 ± 4.548	48.323 ± 4.453	−31.012 ± 2.016
Aldose reductase					
RNT	−45.149 ± 2.951	−15.180 ± 3.971	−60.330 ± 4.869	21.823 ± 2.876	−38.506 ± 3.319
CGA	−45.687 ± 2.949	−30.610 ± 4.368	−76.297 ± 5.030	34.866 ± 8.519	−41.431 ± 7.470
EPT	−41.078 ± 2.944	−34.097 ± 6.898	−75.177 ± 8.385	33.825 ± 5.961	−41.351 ± 3.745
IOR	−60.937 ± 3.431	−29.525 ± 4.654	−90.462 ± 9.270	45.064 ± 7.0224	−45.398 ± 4.568
LGC	−54.243 ± 3.435	−58.854± 7.995	−113.098 ± 8.049	67.995 ± 6.395	−45.102 ± 4.024
RTN	−56.737 ± 6.748	−31.384 ± 5.681	−88.122 ± 12.366	46.000 ± 9.981	−42.122 ± 4.787

ΔEvdW: van der Waals energy, ΔEele: electrostatic energy, ΔEgas: gas-phase free energy, ΔGsol solvation free energy, ΔGbind: total binding free energy, CCT: Cacticin, PDN: Procyanidin, RTN: Rutin, HPS: Hyperoside, DCA: 1,3-dicaffeoxyl quinic acid, LGC: luteolin-7-*O*-beta-d-glucoside, CGA: Chlorogenic acid, EPT: Epicatechin, IOR: Isorhamnetin-3-*O*-rutinoside, Standard drugs [ACB: Acarbose, RNT: Ranirestat].

## Data Availability

The data presented in this study are available in the article.
